# Density Detection of Aligned Nanowire Arrays Using Terahertz Time-Domain Spectroscopy

**DOI:** 10.1186/s11671-016-1551-1

**Published:** 2016-07-19

**Authors:** Wenfeng Xiang, Xin Wang, Yuan Liu, JiaQi Zhang, Kun Zhao

**Affiliations:** State Key Laboratory of Heavy Oil Processing, China University of Petroleum, Beijing, 102249 China; Beijing Key Laboratory of Optical Detection Technology for Oil and Gas, China University of Petroleum, Beijing, 102249 China

**Keywords:** Nanowire density, Terahertz time-domain spectroscopy, Transmitted amplitude, Optical thickness, 42.25.Bs, 68.55.JK, 75.50.Cc, 78.47.+p, 78.67.-n

## Abstract

A rapid technique is necessary to quantitatively detect the density of nanowire (NW) and nanotube arrays in one-dimensional devices which have been identified as useful building blocks for nanoelectronics, optoelectronics, biomedical devices, etc. Terahertz (THz) time-domain spectroscopy was employed in this research to detect the density of aligned Ni NW arrays. The transmitted amplitude of THz peaks and optical thickness of NW arrays was found to be the effective parameters to analyze the density change of NW arrays. Owing to the low multiple scattering and high order of Ni NW arrays, a linear relationship was observed for the transmitted amplitude and optical thickness regarding NW density, respectively. Therefore, THz technique may be used as a promising tool to characterize the density of one-dimensional structures in the large-scale integrated nanodevice fabrication.

## Background

Arrays of one-dimensional (1D) nanostructures such as nanowires (NWs) and nanotubes (NTs) are a very attractive option to be used as building blocks for nanoscale electronic, biosensor, chemical detector, etc. [1–3]. To realize their full potential in applications, however, NWs and NTs must be integrated efficiently into various device architectures. Many factors, such as NW/NT numbers, diameter, and alignment, should be considered in the process of developing large-scale device fabrication. To control the lateral density of NW and NT arrays, since it can influence device performance, is one of the most important challenges. A number of experimental studies have been reported to explore the impact of NW and NT density on device performance in the context of photoelectric response, nanowire electrical transport studies, gas-phase chemical sensing, and field emission [4–7]. The fundamental requirements for these studies are to obtain quantitatively meaningful relationships between the NW and NT density and device properties. Meanwhile, many approaches have been suggested to control the density of NWs [8, 9]. However, these methods only focus on the vertically grown NWs, and the NW density was controlled during the preparation of NWs.

Though there have lots of existing methods such as directed self-assembly, flow-assisted alignment, contact printing, Langmuir–Blodgett technique, and blown bubble methods [10–14] to fabricate the large-scale lateral-aligned NW/NT devices, it is still difficult to precisely control the density of NWs/NTs incorporated into each device. Especially, when the NW arrays have a high density, it is impossible to quantitatively analyze the density distribution of large-scale assembly of NWs and NTs.

Terahertz time-domain spectroscopy (THz-TDS) is a normal and significant THz method based on the THz electric field with time resolution. As a newly developed spectral technique, some spectral features can be used as the standard to qualitatively and quantitatively analyze the material structures and physical properties of tested samples. Ramanandan’s group investigated the oxidation kinetics of nanometer-thick copper films using the in situ THz transmission spectroscopy [15]. Balci’s group investigated the complex refractive index, dielectric function, and conductivity of ZnO NWs using temperature-dependent THz-TDS combined with calculations [16]. In this work, we investigated the relationship between the optical properties and the aligned Ni NW density using the THz-TDS technique. These results indicated that THz-TDS technique was effective to realize the detection of NW density.

## Methods

**Fig. 1:**
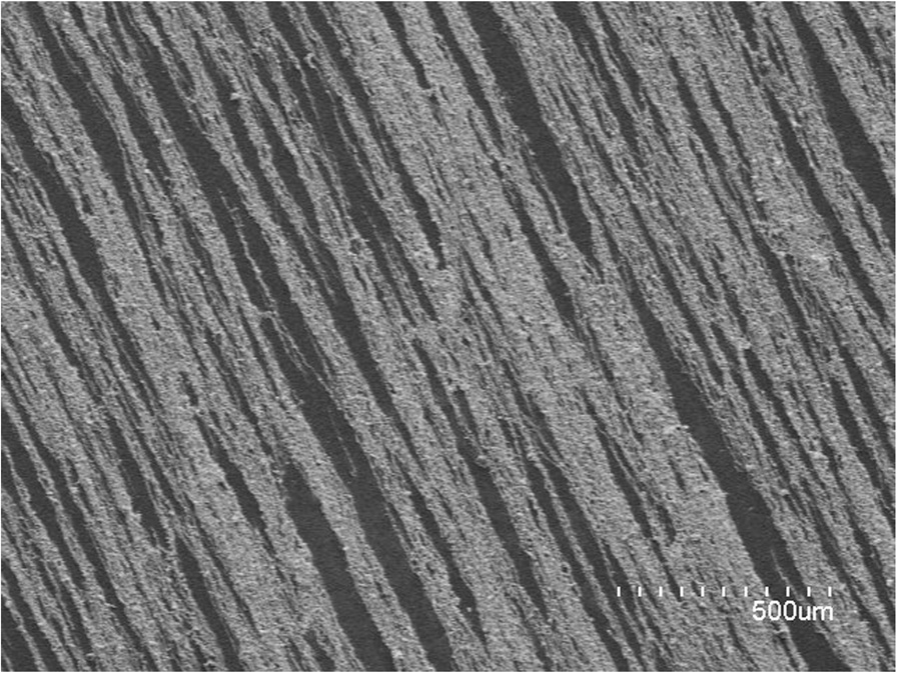
SEM image of Ni NW arrays with NW density of 0.31 × 10^9^ cm^−2^

In order to avoid the light scattering resulted from the disordered arrangement of NWs, the magnetic material of Ni NWs with the similar length and diameter was used and aligned by magnetic field. Nickel nanowires were fabricated by electrochemical deposition into commercially available 50-μm thick alumina filter templates (Anodisc, Whatman, Inc.) [17]. A gold film was deposited on one side of the template to serve as a working electrode. Nickel was deposited from a solution of 20 g/L NiCl_2_ · 6H_2_O and 20 g/L H_3_BO_3_, buffered to pH 3.4 at a potential of 1.0 V. The wires were grown to be ~20 μm in length, as controlled by the deposition time. Next, the AAO membrane was dissolved in a 1-mol/L NaOH solution for 1 h at room temperature. A series of washing steps dilutes the base, and the NWs can be suspended in a number of solvents indefinitely for storage. Owing to a low absorption for the THz wave, the 2-mm thick rectangular polyethylene lamina was used as the substrate. The Ni NWs were aligned in alcohol at 50 °C by magnetic field on the polyethylene substrate with the different density range from 0.31 × 10^9^ cm^−2^ to 2.17 × 10^9^ cm^−2^. The magnetic field intensity was controlled in the range of ~0.6 mT. Figure 1 shows the SEM image of the aligned Ni NW arrays with the NW density of 0.31 × 10^9^ cm^−2^. It can be clearly observed that the horizontally aligned Ni NW arrays spread on the substrate. The mean values of the diameter and length of NWs were calculated by a statistical evaluation of SEM micrographs. Arrays of NWs ~260 nm in diameter and lengths in the 25 μm were obtained.

**Fig. 2:**
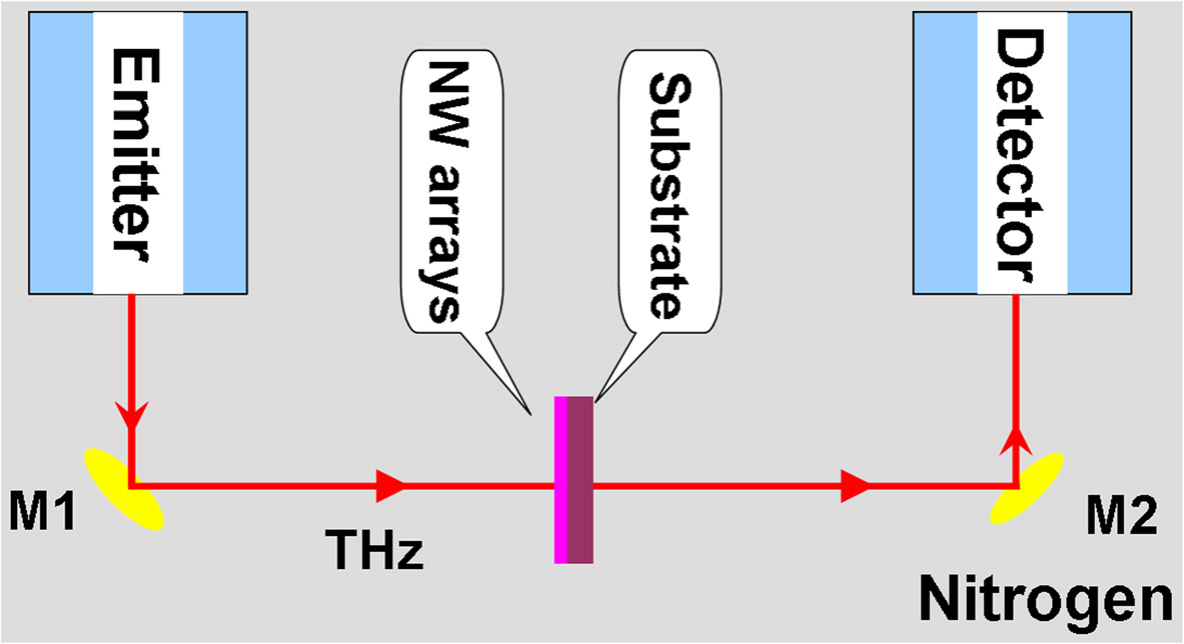
Sketch map of THz-TDS setup

The density of aligned NW arrays was investigated at room temperature by THz-TDS. The measurement system was comprised of a transmission THz-TDS setup and a diode-pump mode-locked Ti:sapphire laser (Maitai, Spectra Physics) from Zomega Terahertz Corporation as shown in Fig. 2. In brief, an 800-nm femtosecond laser beam was split into pump and probe beams. The pump beam (~100 mW) was focused onto the surface of a biased GaAs photoconductive antenna for terahertz generation. THz pulses were focused onto a sample by optical lens, and the THz beam carrying sample’s information met the probe laser beam at the ZnTe crystal in THz dector [18]. All THz-TDS measurements are performed six times for each sample, and the average value was used to analyze the density change of Ni NW arrays. For comparison, the empty polyethylene substrate was used with the same size.

## Results and Discussion

**Fig. 3:**
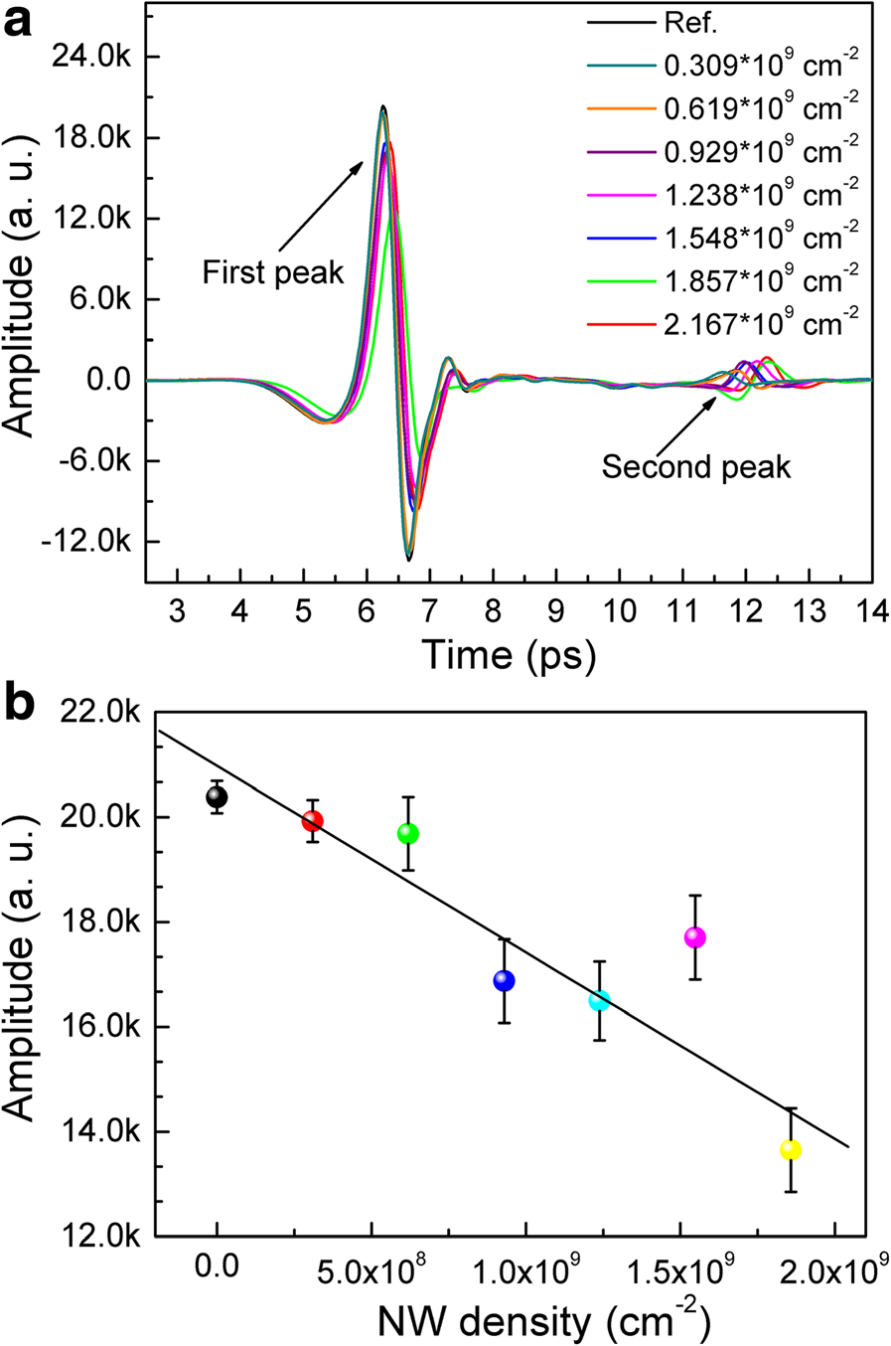
**a** Time-domain spectra of the terahertz wave transmitted through reference and Ni NW arrays and **b** the transmitted amplitude of the first peak as a function of NW density

The reference pulse was obtained by scanning the polyethylene substrate firstly and the THz field amplitude as a function of time after the transmission of the THz pulse through the samples with different NW density as shown in Fig. 3a. It can be seen that from Fig. 3a, there are two peaks occurred in each spectrum, indicating that the THz pulse was reflected within the sample. Moreover, the amplitude of the first peak is higher than that of the second peak owing to the lower reflectance of samples. The amplitude variation of the first peak as the function of NW density is shown in Fig. 3b. Linear relationship is observed for the variation of the peak amplitude regarding the NW density and the slope of the fitting straight line is −0.155. The peak amplitude decreased with the NW density increasing.

**Fig. 4:**
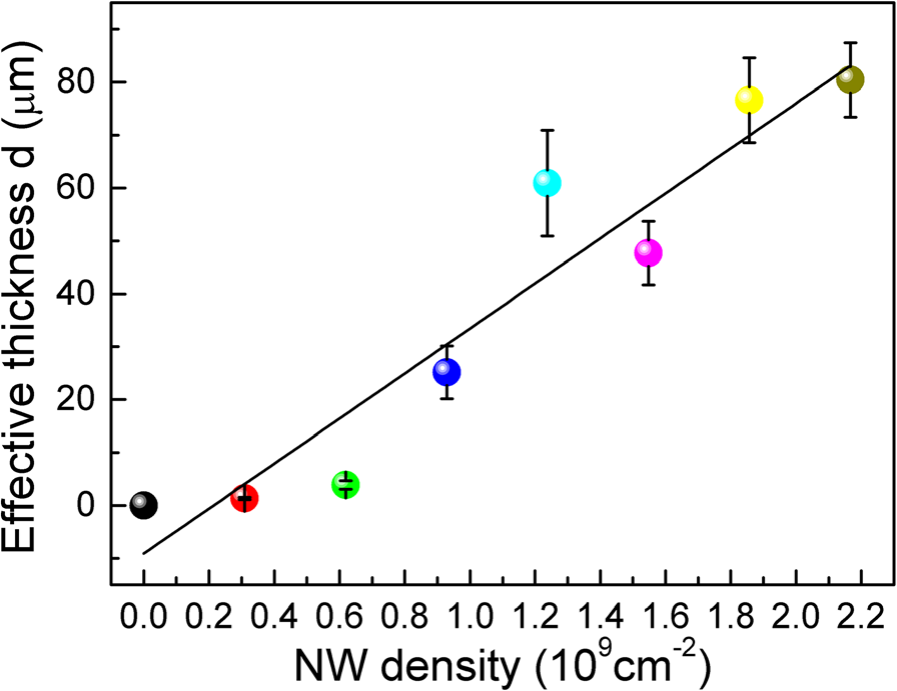
Optical thickness of Ni NW arrays as a function of NW density

In Fig. 3a, it is interesting to note that the location of the first peak has a right shift with NW density increasing, corresponding to the increase of the propagation time through nanowire arrays, i.e., the thickness of nanowire array increased with NW density increasing. In order to investigate the thickness variation of the aligned NW arrays regarding NW density, the optical thickness (*d*_OT_) of nanowire array is defined as the product of the speed of light in air (C) and the propagation time through nanowire array. In Fig. 3a, the first peak is the transmission directly and the second peak is formed that the THz signal through the samples was reflected two times by the back side and front side of the samples continuously. The *d*_OT_ can therefore be calculated by the delay of the signal separating the peaks and is given by

1 $$ {d}_{\mathrm{OT}}=\frac{C\times \left({t}_1-{t}_0\right)}{2} $$

where *t*_1_ and *t*_0_ are the delay of the two peaks in the sample with aligned NWs and in the reference sample, respectively. The *d*_OT_ is plotted as the function of NW density as shown in Fig. 4. A linear relationship between the *d*_OT_ and the NW density is observed, indicating that the effective refractive index of the NW arrays has no obviously change with the NW density increasing.

**Fig. 5:**
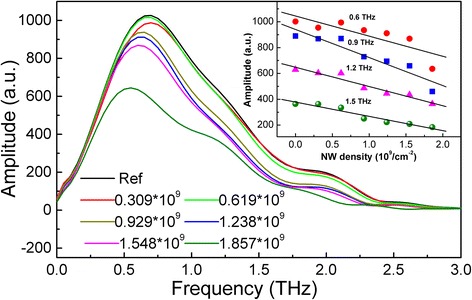
Frequency dependence of amplitude spectra of samples and reference. Inset shows the dependence of transmitted amplitude on the NW density

After the application of fast Fourier transform, the THz frequency-domain spectra (THz-FDS) were calculated in Fig. 5. When the THz frequency is smaller than 0.5 THz, the transmitted amplitude of all samples has no obvious change with NW density increasing. However, the transmitted amplitude decreased regarding NW density when the frequency is larger than 0.5 THz. Inset shows the transmitted amplitude as a function of NW density at selected frequencies of 0.6, 0.9, 1.2, and 1.5 THz. A similar change tendency in the transmitted amplitude at all selected frequencies is clearly observed; i. e., the transmitted amplitude linearly decreased with the NW density increasing.

Usually, scanning electron microscope (SEM) is an appropriate way to describe the density, diameter, and alignment of NWs and NTs in nanodevices. Li et al. have fabricated the Si NW-based field-effect transistor biosensors using nanomanipulation inside a SEM system and the number of NWs in each device is only 1, 4, and 7, respectively [19]. The multiple In_2_O_3_ nanowire devices for gas detection have been reported by Zhang et al., and the SEM image showed that the number of NWs in a device is estimated to be 100~200 [6]. It can be found that SEM system is difficult to detect the exact density of lateral-aligned NWs if the number of NWs in each device is higher than the order of 100 of NW density. Recently, conventional optical technology in the UV-IR region has been used to study the properties of nanostructures. However, the quantitative analysis of the NW density in NW arrays remains difficult. Because the light wavelength is approximately equal to the size of NWs, the nonlinear interaction between light and NWs is strongly dependent on the numbers, shape, and alignment of NWs induced by the multiple scattering effect. In In_2_S_3_ nanostructure arrays, it is found that the springs, screws, and vertical rods have an enhanced absorption compared to zigzags and tilted rods investigated by UV-NIR spectroscopy [20]. Tena-Zaera et al. investigated the optical scattering effect of ZnO NW arrays in visible wavelength range and found that the increase in diameter of NWs induces a considerable redshift in the reflectance maximum [21]. THz-TDS technique was considered to quantitatively estimate the NW density because of its advantages compared to the SEM technique and UV-NIR spectroscopy. The wavelength range of THz signal is from 30 to 1000 μm, which is much larger than the diameter of NWs and the distance between the neighboring NWs. Therefore, the multiple scattering in NW arrays has not been distinctively enhanced with NW density increasing. However, the lower NW density and alignment of NWs will influence the accuracy and measuring resolution of THz-TDS technique.

## Conclusions

In summary, the practicability was demonstrated about the THz-TDS being applied to quantitatively measure the density of aligned NW arrays. The density variation of Ni NW arrays as the function of the transmitted amplitude and optical thicknesses of all samples was analyzed, and the linear relationship was found. It is indicated that THz-TDS represents a powerful tool for the fabrication of 1D nanoscale devices in future.
